# Constituents of stable commensal microbiota imply diverse colonic epithelial cell reactivity in patients with ulcerative colitis

**DOI:** 10.1186/s13099-024-00612-0

**Published:** 2024-03-23

**Authors:** Ruta Inciuraite, Rolandas Gedgaudas, Rokas Lukosevicius, Deimante Tilinde, Rima Ramonaite, Alexander Link, Neringa Kasetiene, Mindaugas Malakauskas, Gediminas Kiudelis, Laimas Virginijus Jonaitis, Juozas Kupcinskas, Simonas Juzenas, Jurgita Skieceviciene

**Affiliations:** 1 Institute for Digestive Research, Academy of Medicine, Lithuanian University of Health Sciences, Kaunas, Lithuania; 2 Department of Gastroenterology, Academy of Medicine, Lithuanian University of Health Sciences, Kaunas, Lithuania; 3 Department of Gastroenterology, Hepatology and Infectious Diseases, Otto-von-Guericke University Hospital Magdeburg, Magdeburg, Germany; 4 Department of Food Safety and Quality, Academy of Veterinary, Lithuanian University of Health Sciences, Kaunas, Lithuania; 5 Institute of Biotechnology, Life Sciences Center, Vilnius University, Vilnius, Lithuania

**Keywords:** Ulcerative colitis, Gut microbiota, *Escherichia coli*, *Phocaeicola vulgatus*, Colonic epithelial organoids, Colonic epithelial barrier, Crosstalk

## Abstract

**Background:**

Despite extensive research on microbiome alterations in ulcerative colitis (UC), the role of the constituent stable microbiota remains unclear.

**Results:**

This study, employing 16S rRNA-gene sequencing, uncovers a persistent microbial imbalance in both active and quiescent UC patients compared to healthy controls. Using co-occurrence and differential abundance analysis, the study highlights microbial constituents, featuring *Phocaeicola*, *Collinsella*, *Roseburia*, *Holdemanella*, and *Bacteroides*, that are not affected during the course of UC. Co-cultivation experiments, utilizing commensal *Escherichia coli* and *Phocaeicola vulgatus*, were conducted with intestinal epithelial organoids derived from active UC patients and controls. These experiments reveal a tendency for a differential response in tight junction formation and maintenance in colonic epithelial cells, without inducing pathogen recognition and stress responses, offering further insights into the roles of these microorganisms in UC pathogenesis. These experiments also uncover high variation in patients’ response to the same bacteria, which indicate the need for more comprehensive, stratified analyses with an expanded sample size.

**Conclusion:**

This study reveals that a substantial part of the gut microbiota remains stable throughout progression of UC. Functional experiments suggest that members of core microbiota – *Escherichia coli* and *Phocaeicola vulgatus –* potentially differentially regulate the expression of tight junction gene in the colonic epithelium of UC patients and healthy individuals.

**Supplementary Information:**

The online version contains supplementary material available at 10.1186/s13099-024-00612-0.

## Background

Ulcerative colitis (UC) is a complex, chronic inflammatory disorder, characterized by periods of relapse and remission, often leading to significant morbidity and reduced quality of life [1]. While the exact etiology of UC remains elusive, emerging evidence points towards an important role for the gut microbiota in disease pathogenesis [2]. The interplay between the host and its microbial inhabitants is known to be a crucial factor of intestinal homeostasis, which is commonly impaired in UC [3].

In this study we aim to investigate the relationship between gut microbiota dynamics as well as epithelial cell response to commensal bacteria in patients with UC and healthy individuals. Specifically, we explore the alterations as well as consistencies in the composition of the gut microbiota in the individuals afflicted with UC. Additionally, we focus on how co-cultivation of stable, rather than altered, predominantly commensal bacteria (such as *Escherichia coli* and *Phocaeicola vulgatus*) [4, 5] with healthy or UC patient-derived colonic epithelial organoids affect host gene expression responsible for pathogen recognition, tight junction regulation and stress stimuli indication, and how this response differs between UC-afflicted and healthy colonic epithelial cells. Understanding the intricate crosstalk between the host response and the consistently resident microbiota holds the potential to uncover novel insights into the mechanisms underlying UC pathogenesis.

In this context, we present a comprehensive analysis of gut microbiota profiles in active and quiescent UC patients as well as healthy individuals, shedding light on altered and stable microbiota. Importantly, we focus on the bacteria that remain unaltered after undergoing the reduction of diversity during the pathogenesis of UC. Furthermore, we delve into the putative functional consequences of these unaltered and predominantly commensal bacteria to discern their potential implications for disease progression, including their capacity to trigger UC relapses.

## Methods

### Study samples

Study subject recruitment was conducted at the Department of Gastroenterology, Lithuanian University of Health Sciences (Kaunas, Lithuania) during the period of 2020–2022. The study was approved by the Kaunas Regional Biomedical Research Ethics Committee (Protocol No. BE-2-31) and all subjects signed written informed consent to participate in the study. All procedures were performed in accordance with relevant guidelines and regulations. Colonic biopsies were obtained from patients with a previously established diagnosis of UC (based on clinical, endoscopic, and histological examinations). Individuals without inflammatory, oncological, or other gastrointestinal diseases were enrolled in the study as controls. UC patients underwent colonoscopy procedures either because of a disease flare or for screening purposes, while control individuals underwent colonoscopy procedure through colorectal cancer screening program. The study included two cohorts of samples (Table 1). UC patients were subgrouped based on endoscopic Mayo score (score of 0–1 was considered mild disease (healed mucosa), 2 reflected moderate severity of UC, and 3 was considered as an indicator for severe UC (with spontaneous bleeding and ulcerations in the colon) [6]. Individuals with an endoscopic Mayo score > 1 were classified as active UC patients, while those with endoscopic Mayo score ≤ 1 were considered as a quiescent UC (in remission) group. The age and sex of individuals did not differ significantly between patient groups of each cohort (cohort 1 and 2).

**Table 1:** Demographic and clinical characteristics of the study subjects

	Cohort 1, *n* = 72			Cohort 2, *n* = 17	
	Control, *n* = 25	Active UC, *n* = 27	Quiescent UC, *n* = 20	Control, *n* = 8	Active UC, *n* = 9
**Age**					
Mean ± SD	40.9 ± 13.2	43.3 ± 17.3	45.8 ± 15.3	56.9 ± 7.3	44.2 ± 15.9
**Sex, n (%)**					
Female	3 (50.0)	3 (50.0)	3 (43.0)	4 (50.0%)	4 (44.4%)
**Endoscopic Mayo score**					
Min-max	-	2–3	0–1	-	2–3

SD – standard deviation, UC – ulcerative colitis

### Nucleic acid extraction

For gut microbiota analysis, nucleic acids were extracted from fecal samples using the AllPrep PowerFecal DNA/RNA kit (Qiagen) following the manufacturer’s protocol. In brief, up to 200 mg of fresh-frozen fecal samples were lysed using chemical and mechanical homogenization and DNA was eluted into 30 µl of elution buffer. For colonic epithelial cell gene expression analysis, intestinal monolayer cultures were processed using AllPrep DNA/RNA Micro Kit (Qiagen). Cells were lysed and homogenized chemically, using denaturing guanidine isothiocyanate-containing buffer. Purified RNA was eluted into 14 µl of RNAse and DNAse-free water. Purity and concentration of extracted nucleic acids were evaluated using Qubit 4 (Invitrogen) fluorometer and respective assay kits.

### 16S rRNA-gene library preparation and sequencing

The isolated DNA underwent amplification with the specific primer pair set 27F 5’-AGAGTTTGATCCTGGCTCAG-3’ and 338R 5’-TGCTGCCTCCCGTAGGAGT-3’, using dual-indexing during the PCR process. Cycling conditions: 1 × 98 °C 30 s.; 34 × 98 °C 9 s., 50 °C 1 min., 72 °C  20 s.; 1 × 72 °C 10 min; 1 × 10 °C ∞. Purification and normalization of the PCR products were carried out using the Invitrogen SequalPrep Normalization Plate Kit (Thermo Fisher Scientific). After the preparation, 16S rRNA gene sequencing was conducted on the Illumina MiSeq platform in accordance with the manufacturer’s instructions, utilizing MiSeq Reagent Kit v3 (2 × 300 bp) (Illumina).

### 16S rRNA-gene sequencing data analysis

The obtained sequencing data were processed into amplicon sequencing variants and taxonomically annotated against the RDP v18 database [7] using the ‘DADA2’ (V.1.10) [8] software package in R, following the DADA2 workflow. Specifically, reads were truncated to 200 base pairs for forward and 150 base pairs for reverse using the truncLen parameter, while the maximum number of expected errors (maxEE parameter) was set to 3 for both directions. Additionally, trimming of the first 5 bases from both forward and reverse reads (trimLeft parameter) was performed to enhance overall quality, with primer sequences already removed from the fastQ files. The maxN parameter was set to 0, indicating the exclusion of reads containing ambiguous base calls (N’s). Reads were truncated at the first instance of a quality score equal to or lower than 5 using the truncQ parameter. These parameter configurations were chosen to ensure the retention of high-quality reads while effectively filtering out artifacts and low-quality regions. Rarefaction was used as a measure of normalization, with all samples rarefied to 22,032 reads per sample. Rare taxa, defined as ASVs with fewer than 10 counts and present in less than 10% of total samples were filtered before performing α-diversity, β-diversity and compositional analyses. Alpha diversity was assessed using the Chao1, Simpson and Shannon index, while Bray Curtis dissimilarity on taxa relative abundances was used as a measure of β-diversity. Permutational analysis of variance (PERMANOVA) within the vegan package was employed to identify significant changes in Bray-Curtis dissimilarity. For core microbiome analysis, a minimum relative abundance of 0.1% in at least 50% of samples was applied. Differential abundance analysis was conducted on the taxa count matrix utilizing the Wilcoxon rank-sum test. This analysis focused only on taxa that had a minimum count of 10 and appeared in more than 20% of the samples. The P values obtained from the Wilcoxon rank-sum test underwent Benjamini-Hochberg (BH) correction to control the false discovery rate. A corrected P value (BH adjusted P_Wilcoxon_) threshold of 0.05 was set to determine statistical significance in the differential abundance analysis. Compositional plots were generated using microViz package [9].

### Establishment and expansion of 3D colonic epithelial organoids

3D undifferentiated colonic epithelial organoids from adult intestinal stem cells were established and cultured according to the protocol of IntestiCult Organoid Growth Medium (Human) (OGMH) (StemCell Technologies) with slight adjustments. Briefly, colon biopsies were minced and digested using Gentle Cell Dissociation reagent (StemCell Technologies). To further isolate colonic crypts from tissue homogenate, samples were vigorously pipetted in cold DMEM/F-12 (supplemented with 1% BSA and 15 mM HEPES) medium, passed through a 70 μm pore filter and centrifuged. Isolated colonic crypts were mixed with extracellular matrix (Matrigel Matrix Phenol Red-free, LDEV-Free (Corning)). The volume of 50 µl of crypt-Matrigel mixture was used to form domes in a 24-well cell culture plate. Colon organoids were cultured in OGMH medium supplemented with penicillin/streptomycin (100 µg/ml) (Gibco). Medium also contained RHO/ROCK signaling pathway inhibitor Y-27,632 (10 µM) (Stemcell Technologies) for the first two days of culturing. Colonic epithelial organoids were incubated at 37 °C with 5% CO_2_. Undifferentiated 3D organoids were microscopically evaluated using ZEISS Axio Observer 7 and ZEISS ZEN 3.1 (blue edition) software (ZEISS). The primary splitting of colonic epithelial organoids was performed after 1–2 weeks from culture establishment. Subsequent passaging of cultures was performed every 7–10 days depending on the maturity of organoids (usually, 7–10 days post-passage).

### Establishment of colonic epithelial cell monolayers

Human colonic epithelial cell monolayers were established from expanded 3D colonic epithelial organoids in 24-well cell culture plates (Falcon). Briefly, each well of the cell culture plate was coated with Collagen I, Rat tail (Gibco) (≈ 5 µg/cm^2^) for 2 hours at 37ºC, then washed with PBS. Simultaneously, undifferentiated 3D colonic epithelial organoids were reduced into single cell suspensions. Organoids were disrupted by adding TrypLE Express (Gibco) supplemented with Y-27632 (StemCell Technologies) and incubating suspensions at 37ºC for 10 min. The suspension was pipetted every 5 min to ensure the appropriate cell separation. TrypLE Express was blocked by addition of equal volume of DMEM/F-12 (StemCell Technologies) and suspension was centrifuged at 400 x*g* for 5 min. Pellet was resuspended in DMEM/F-12, passed through a 40 μm cell strainer and centrifuged again. Colonic epithelial cells were resuspended in IntestiCult OGMH (StemCell Technologies) supplemented with penicillin/streptomycin (100 µg/ml) (Gibco) and Y-27632 (10 µM) (Stemcell Technologies) and plated on the Collagen I-coated wells. The number of 5 × 10^5^ cells was used per well for seeding monolayers. Monolayers were incubated at 37 °C with 5% CO_2_. The growth of 3D organoid-derived colonic epithelial cell monolayers was monitored under the microscope every day. Cell culture medium (IntestiCult OGMH supplemented with penicillin/streptomycin and Y-27632) was changed every 2–3 days until monolayer reached 100% confluency. Then, culturing medium was changed into cell differentiation medium (IntestiCult Organoid Differentiation Medium (Human) (ODMH) (StemCell Technologies)) supplemented with DAPT (5 µM), penicillin/streptomycin (100 µg/ml) (Gibco) and Y-27632 (10 µM) for 5 days to induce stem cell transition into specialized colonic epithelial cell types. Medium change was performed every 2 days. Monolayers were microscopically evaluated using ZEISS Axio Observer 7 and ZEISS ZEN 3.1 (blue edition) software (ZEISS).

### Immunofluorescence microscopy

The cellular and structural composition of the established patient organoid-derived differentiated colonic epithelial cell monolayers was evaluated by immunofluorescence microscopy. First, monolayers were formed on 8-well format Collagen I-coated Nunc Lab-Tek II Chamber Glass slides (Thermo Scientific) and grown until full confluency and then differentiated as described above. Further, monolayers were fixed by incubating them in 4% paraformaldehyde (Sigma-Aldrich) solution for 30 min at RT. Further, colonic epithelial cell monolayers were permeabilized by using 0.5% Triton-X (Sigma-Aldrich) solution and blocked with 2% BSA blocking solution. Finally, conjugated monoclonal antibodies were diluted in antibody dilution solution (dilution ratio 1:50 − 1:500), applied to the processed monolayers and incubated for 60 min at RT. Conjugated antibodies for (i) tight-junction marker (Anti-ZO-1-Alexa Fluor 555 (MA3-39100-A555, Invitrogen)), (ii) proliferating cell marker (Anti-ki67-Alexa Fluor 488 (ab206633, Abcam)), differentiated/specialized cell markers (for Goblet cells, colonocytes, enteroendocrine cells) (Anti-Mucin2-Alexa Fluor 555 (bs-1993R-A555, Biocompare), anti-Cytokeratin 20-Alexa Fluor 488 (ab275988, Abcam), anti-Chromogranin A-Alexa Fluor 488 (ab199192, Abcam), respectively) were used. Hoechst 33342 (Invitrogen) was used as a counterstain for cell nuclei. All images were acquired with ZEISS Axio Observer 7 inverted fluorescence microscope using 5x and 10x objectives and analyzed by ZEISS ZEN 3.1 (blue edition) software (ZEISS).

### Bacteria cultivation and preparation for co-culturing

Reference strains used for the tests were *Escherichia coli* ATCC 25,922 (Thermo Scientific) and *Phocaeicola vulgatus* ATCC 8482 (ATCC). Before assembling the co-culture system, bacteria were kept at -80ºC in Brain Heart Infusion Broth with glycerol (30%). At first, bacteria were inoculated on agar. Specifically, Trypton Soy Agar (TSA) (Sigma-Aldrich) was used for *Escherichia coli*, while Trypton Soy Agar supplemented with Defibrinated Sheep blood (5%) (Liofilchem) was used for *Phocaeicola vulgatus*. Both strains were cultivated for 24 h at 37ºC; *Escherichia coli* were cultured under aerobic conditions, while anaerobic conditions were used for *Phocaeicola vulgatus*. Bacterial suspensions were prepared using phosphate-buffered saline solution (Invitrogen).

### Colonic epithelial cell and bacteria co-culturing

Differentiated patient-derived colonic epithelial cell monolayers and two bacterial strains - *Escherichia coli* and *Phocaeicola vulgatus* - were used to establish a co-culture systems. Monolayers cultured without bacteria were used as control samples. First, to assemble co-cultures, cell differentiation medium was removed, and epithelial cell monolayers were washed twice with 500 µl of pre-warmed D-PBS (StemCell Technologies). Bacterial suspensions were centrifuged, and pellet was resuspended in a differentiation medium without antibiotics (IntestiCult ODMH supplemented with DAPT (5 µM) and Y-27632 (10 µM)). 2 × 10^6^ of bacteria (*Escherichia coli* or *Phocaeicola vulgatus)* were added into respective wells with epithelial cell monolayers and co-cultures were incubated for 2 h at 37ºC with 5% CO_2_. After incubation, cell culture medium containing bacteria was discarded, epithelial cell monolayers were washed twice with 500 µl of D-PBS. Then, 500 ul of pre-warmed IntestiCult ODMH (StemCell Technologies)) supplemented with DAPT (5 µM), penicillin/streptomycin (100 µg/ml) (Gibco) and Y-27632 (10 µM) was added into each well and monolayers were cultured for additional 24 h at 37ºC with 5% CO_2_. After incubation, monolayers were washed with 500 µl of D-PBS and lysed using 350 µl of RLT Plus buffer (supplemented with 1% of β-mercaptoethanol) (Qiagen). Lysates were stored at -80ºC until further use for nucleic acid extraction.

### Targeted gene expression analysis using RT-qPCR

To evaluate the expression of *TLR4*, *ZO1*, *HSPA1A* and *HSPB1* genes in patient organoid-derived colonic epithelial cell monolayers, total RNA from these samples was reverse transcribed using High-Capacity cDNA Reverse Transcription Kit (Applied Biosystems). Up to 500 ng of total RNA was used per reaction to synthesize first strand cDNA. Further, the measurement of gene expression was based on SYBR Green chemistry by using SYBR Green PCR Master Mix (Applied Biosystems) and pairs of gene-specific primers (final concentration of each primer − 300 nM). Primers used for amplification and amplicon size are listed in Table 2. Cycling conditions: 1 × 95 °C 10 min.; 40 × 95 °C 15 s., 60 °C 1 min. Analysis was performed on the 7500 Fast Real-Time PCR System (Applied Biosystems). The amount of 4 ng of template DNA was used for each reaction. The cycle threshold (C_T_) values of genes-of-interest were normalized to the value of *ACTB* reference gene. All the procedures were performed in accordance with the manufacturer’s protocol and recommendations.

**Table 2:** Primers used for targeted gene expression analysis

Gene	Transcript ID	Forward primer sequence (5’-3’)	Reverse primer sequence (5’-3’)	Amplicon size, bp
*ACTB*	NM_001101.5	GGACTTCGAGCAAGAGATGG	TGTGTTGGCGTACAGGTCTTTG	229
*TLR4*	NM_138554.5	ATATTGACAGGAAACCCCATCCA	AGAGAGATTGAGTAGGGGCATTT	300
*HSPA1A*	NM_005345.6	CCCCACCATTGAGGAGGTAG	ACATTGCAAACACAGGAAATTGA	124
*HSPB1*	NM_001540.5	AAGCTAGCCACGCAGTCCAA	CGACTCGAAGGTGACTGGGA	51
*ZO1*	NM_003257.5	CGGTCCTCTGAGCCTGTAAG	GGATCTACATGCGACGACAA	371

### Statistical analysis

Statistical gene expression analysis was performed using R Studio software (version 4.3.2). Data distribution was determined using the Shapiro-Wilk test, gene expression differences were analyzed using the Wilcoxon rank-sum test. The difference between the values was considered significant when *P* < 0.05.

## Results

### UC harbors reduced diversity of gut microbiota

**Fig. 1:**
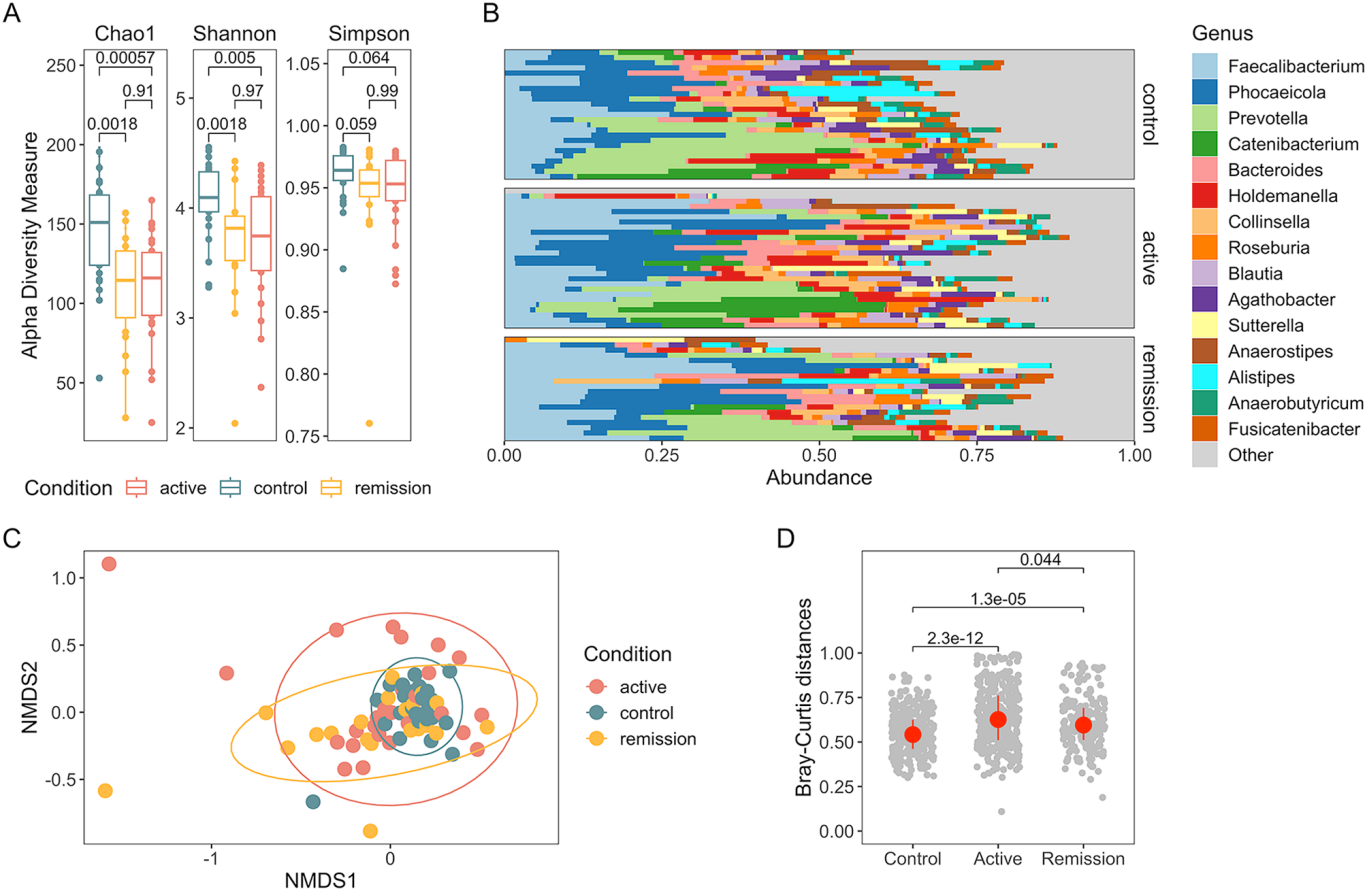
Composition of microbiome in active and quiescent (remission) UC compared to healthy controls. (**A**) Boxplots representing median and Q1-Q3 values of alpha diversity metrics. Numbers indicate p value between the groups assessed by Wilcoxon rank-sum test. (**B**) Bar plots displaying relative abundances of top 15 most abundant genera in the study cohort, genera not in the top 15 are marked as Other. (**C**) Non-metric multidimensional scaling (NMDS) plot of complete dataset based on Bray-Curtis distances showing compositional differences between groups. (**D**) Scatter plot comparing in-between sample similarity in respective condition groups based on Bray-Curtis dissimilarity index

To resolve the composition of gut microbiota, we performed 16S rRNA-gene sequencing of fecal microbiomes in active and quiescent UC as well as in healthy individuals. To ensure data quality, we rigorously preprocessed sequencing reads by implementing strict quality control parameters (see Methods and Supplementary Table S1). Bacterial diversity (α-diversity), assessed by Chao1, Shannon and Simpson diversity indices, indicated that control individuals exhibited significantly greater species richness and diversity in comparison to those with active or quiescent UC (Fig. 1A). Interestingly, there were no differences between UC disease activity states, showing that UC patients, that are in remission, already harbor less diverse microbiomes than healthy individuals (Fig. 1B). Similarly, microbial community clusters (β-diversity), evaluated using the Bray-Curtis dissimilarity index, significantly differed between control subjects and patients with active or quiescent UC (P_PERMANOVA_ = 0.008 (R-squared value = 0.047) and P_PERMANOVA_ = 0.01 (R-squared value = 0.052), respectively). Notably, no significant clusters were identified among different disease activity states (P_PERMANOVA_ = 0.49) (Fig. 1C). Reflecting similar patterns, in-between sample dissimilarity also assessed by Bray-Curtis dissimilarity index showed that samples from control subjects had significantly higher in-between sample similarity (mean 0.548 ± 0.118) than patients with active disease (mean 0.640 ± 0.168) and patients in remission (0.607 ± 0.148). Quiescent UC patients also bore significantly higher similarity than patients with active UC (Fig. 1D).

Taken together, the results show decreased diversity and altered microbiota not only in the active, but also in quiescent UC patients compared to healthy controls.

### Common core microbiome among UC and healthy controls

**Fig. 2:**
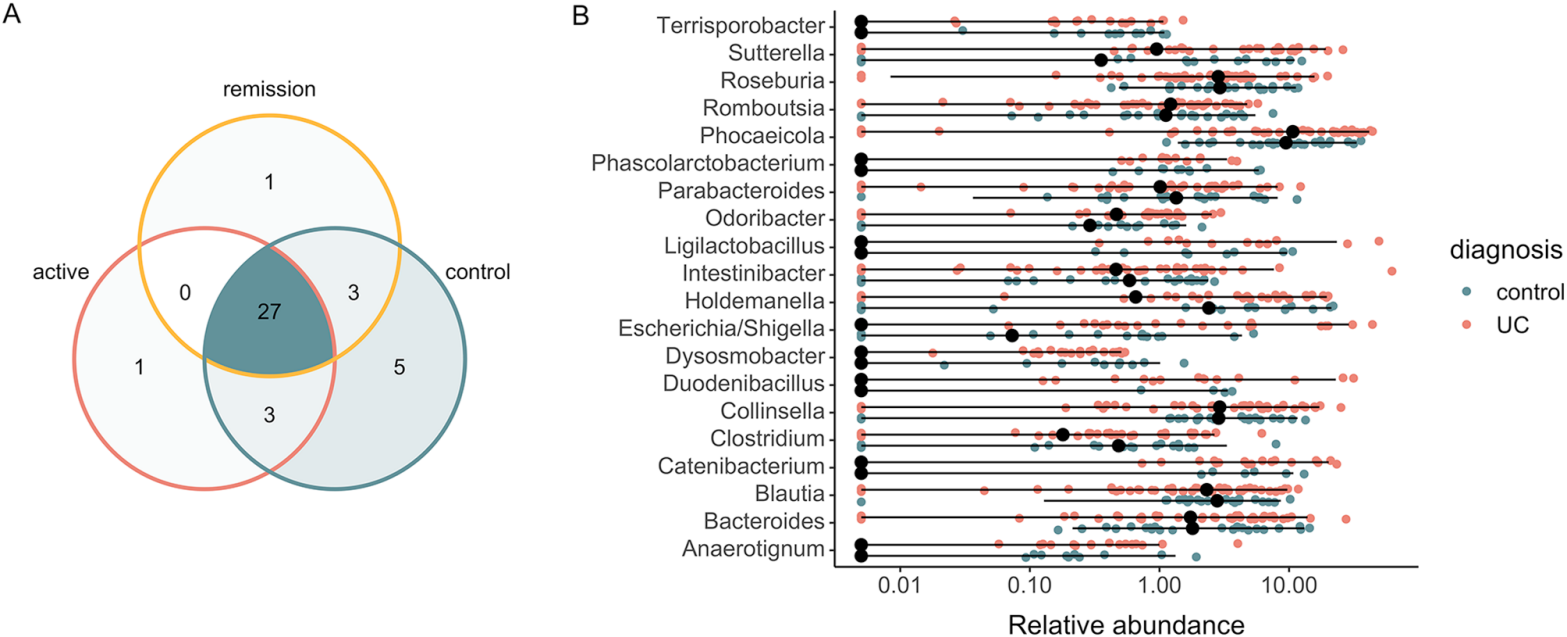
Fecal core microbiome among active and quiescent (remission) UC patients and healthy controls. (**A**) Venn diagram of exclusive and shared core taxa at genus level (minimum prevalence − 0.1% in at least 20% of samples in each group) based on respective condition. (**B**) Most constituently abundant (*N* = 20) genera between healthy controls and active UC groups

To investigate not only the altered taxa, but more importantly, the stable (common) core microbiota across different stages of UC and healthy individuals, multiple analyses, including co-occurrence and differential abundance were performed. In total, 27 genera (such as *Intestinibacter*, *Phocaeicola*, *Ligilactobacillus*, *Bacteroides*, *Escherichia/Shigella*, etc.) were identified to be shared and consistently present in the feces of active and quiescent UC patients as well as healthy controls (Fig. 2A). Compared to healthy individuals, UC patients contained 5 genera (such as *Alistipes*, *Mediterraneibacter*, *Paraprevotella*, etc.), that showed statistical significance (BH adjusted P_Wilcoxon_ < 0.05) in relative abundance, while 35 genera were present at similar levels (Fig. 2B and Supplementary Table S2). Among the commonly present and non-altered taxa, the most abundant ones were *Phocaeicola*, *Collinsella*, *Roseburia*, *Holdemanella* and *Bacteroides* (Supplementary Table S2), and most of which are known to be predominantly commensal bacteria as well as considered as a core microbiome to sustain intestinal homeostasis [10].

Collectively, the results indicate that a substantial portion of the gut microbiota is consistently present and remains unchanged throughout the pathogenesis of UC. It is meaningful to acknowledge that the stability of these bacteria might be important in understanding the condition.

### UC patient-derived colonic epithelial cells show diverse reactivity to constituent bacteria

**Fig. 3:**
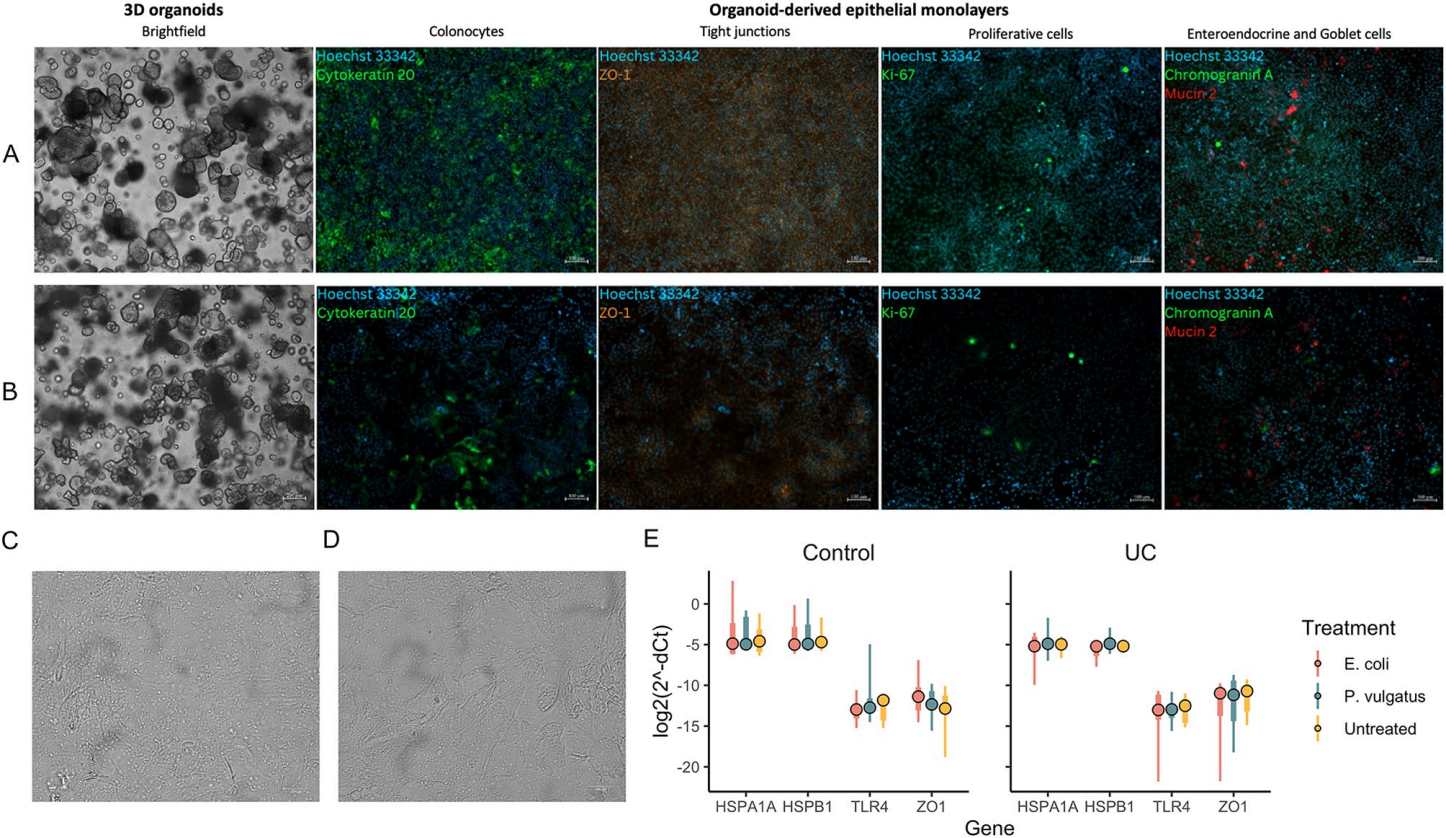
3D colon organoids and organoid-derived colonic epithelial monolayers resemble the typical appearance of colonic epithelium and empower co-cultivation with commensal bacteria. **(A-B)** Representative pictures of 3D colonic epithelial organoids (colonoids) and cellular composition of organoid-derived monolayers of control individual **(A)** and patient with active UC **(B)**. Hoechst 33,342 (blue) was used in all cases as a counterstain for cell nuclei. Areas of proliferation are identified by Ki67 (green) expressing proliferating cells. Epithelial barrier integrity is defined by detection of tight junction protein ZO-1 (orange). Absorptive colonocytes are defined by positive Cytokeratin 20 (green) staining. Mucin-producing Goblet cells are identified by positive Mucin 2 (red) staining. Hormone-producing enteroendocrine cells are defined by positive Chromogranin A (green) staining. **(C-D)** Representative pictures of colonic epithelial monolayers co-cultured with *Escherichia coli***(C)** and *Phocaeicola vulgatus***(D)**. **(E)** Expression analysis of marker genes (x-axis), representing host response to pathogen recognition (*TLR4*), tight junction regulation (*ZO1*) and stress stimuli indication (*HSPA1A* and *HSPB1*). Expression estimates (Ct) were normalized to *ATCB* (delta-Ct) and were inverted (as log_2_(2^^−deltaCT^)) to recapitulate direction of the effect

To gain some functional insights on the stable core gut microbiome and how colonic epithelial cells respond and react to their presence as well as how this response is different in the cells from healthy and UC-afflicted individuals, co-cultivation experiments were performed. Specifically, we employed patient-derived 3D colonic epithelial organoid technology to further establish organoid-derived epithelial monolayers (2D cultures) from healthy (*N* = 8) and UC-afflicted (*N* = 9) individuals (Fig. 3A-B). These intestinal cell monolayers were then used for co-cultivation with *Escherichia coli* and *Phocaeicola vulgatus* (Fig. 3C-D). The selection of these bacteria was based on the fecal microbiota sequencing results showing that these species belonged to the ones of the most constituent genus among healthy and UC-afflicted individuals. Host response to bacteria was evaluated using targeted gene expression analyses of markers responsible for pathogen recognition (*TLR4*), tight junction regulation (*ZO1*) and stress stimuli indication (*HSPA1A* and *HSPB1*). Upon examining the marker gene expression, we observed insightful trends in the exposed cultures. First, neither *E. coli*, nor *P. vulgatus* were recognized as pathogens or induced stress response as assessed from the expression of *TLR4, HSPA1A* and *HSPB1* in the epithelial cells of healthy controls and UC patients (Fig. 3E). Interestingly, there was a trend for an increase in *ZO1* expression in control-derived monolayers (log_2_FC = 1.8 [*E. coli*] and log_2_FC = 0.85 [*P. vulgatus*]), while a trend for decrease was observed in UC-afflicted colonic epithelial cells compared to mock (untreated) versus co-cultured cells (log_2_FC = -1.25 [*E. coli*] and log_2_FC = -0.47 [*P. vulgatus*]), suggesting a putative differential response in tight junction formation and integrity, which is suggestively reduced in the UC-derived epithelial cells (Fig. 3E). However, we could not identify any statistically significant changes in response to bacteria between UC and controls due to a relatively small sample size and huge patient-specific variation in response to co-cultivation with bacteria, even in the control individuals. For example, an average variance of normalized gene expression values between biological groups were reaching up to 11.4 and 18.8, respectively, for control- and UC-derived organoids co-cultured with *P. vulgatus* (Supplementary Table S3).

To summarize, the results show a tendency to differential response to *E. coli* and *P. vulgatus* in tight junction formation between control- and UC patient-derived colonic epithelial cell monolayers. Results also show that a host response to intestinal bacteria is very patient-specific, and that patients’ colonic epithelial cells react very differently to the same bacteria.

## Discussion

Typically, the changes in the gut microbiome are deemed as functionally relevant, while unaltered and consistent taxa are often overlooked as irrelevant. For example, several studies have focused on microbiome alterations and their putative functional implications rather than delving into characteristics of the preserved microbiome [11–15]. Indeed, dysbiotic changes in microbiota composition are certainly important and have been shown to provide decisive insights into the pathogenesis of UC as well as being utilized to monitor disease activity or even to treat patients (using such procedures as fecal microbial transplantation) [16]. However, it is still unknown whether the so-called core microbiome, which remains stable amid the ongoing reduction in diversity during UC pathogenesis, can potentially trigger or contribute to the relapse of the disease. To get more insights of the enduring microbial constituents, in this study we used 16S rRNA-gene sequencing to determine the composition of gut microbiota in UC as well as constituent genera. Additionally, we explored the impact of commensal bacteria from these unaltered genera, specifically, *Escherichia coli* and *Phocaeicola vulgatus*, on the colonic epithelial cells of healthy individuals and patients with UC through co-cultivation experiments.

Our findings on microbial composition align with previous research, indicating a substantial decrease in microbial diversity in UC patients when compared to healthy controls [14, 17]. However, our study extends this understanding to include alterations in quiescent patients, suggesting a persistent imbalance in microbial composition even during seemingly inactive phases of the disease. This observation supports the results of Öhman et al., who demonstrated in a follow-up study that the gut microbiota of UC patients remains remarkably stable regardless of disease stage, activity, or treatment escalation [18]. Our next focus was to establish the so-called stable core microbiome among the UC patients and control individuals. For this purpose, we combined co-occurrence and differential abundance analysis (to omit differentially abundant), and have identified the most consistent genera, including *Phocaeicola*, *Collinsella*, *Roseburia*, *Holdemanella* and *Bacteroides*. Although we identified *Phocaeicola*, *Bacteroides*, and *Roseburia* genera as constituent, there are studies showing their altered abundance in UC [15, 19, 20]. This might be due to various reasons, including demographics and diet habits of the enrolled individuals, since it is known that the major factor defining microbiome is environment [20]. Generally, it is rather challenging to compare our results from this analysis with other studies, primarily due to the predominant focus of other studies on describing microbiome alterations rather than uniformity. Although our primary focus was on the stable core microbiota, it is noteworthy that our identified differentially abundant genera (*Alistipes, Mediterraneibacter, Paraprevotella*) were previously shown to be also altered in IBD by other authors [21–23]. Further, we have selected two bacteria, namely, *Escherichia coli* and *Phocaeicola vulgatus* (formerly, *Bacteroides vulgatus*), which belong to our identified stable core genera among UC patients and control individuals. The selection of these two specific bacteria was mainly based on the availability of techniques and validated protocols for maintaining bacteria species in culture [24, 25] well as in the co-culture with colonic epithelial cells [26, 27]. Moreover, both *Escherichia coli* and *Phocaeicola vulgatus* are known as life-long highly abundant residents of normal intestinal microbiota in humans [28–30]. Therefore, to finally evaluate if these bacteria can trigger different responses in UC patients than in controls, we performed co-cultivation experiments using intestinal organoid monolayers derived from tissue-resident adult stem cells. Precisely, we evaluated the changes in gene expression of established markers for pathogen recognition (*TLR4*) [20], tight junction regulation (*ZO1*) [31] and stress stimuli indication (*HSPA1A* and *HSPB1*) [32]. Our investigation into the interaction between the gut microbiota and colonic epithelial cells revealed intriguing insights into host responses. We observed a trend to a differential response in tight junction maintenance (based on *ZO1* gene expression) between control- and UC-derived epithelial monolayers co-cultivated with both *Escherichia coli* and *Phocaeicola vulgatus*. Even though controversionally, both bacteria were previously described to be functionally relevant in the pathogenesis of the UC. Mills et al. has shown that proteases released by *Phocaeicola vulgatus* are involved in the dysfunction of epithelial barrier during UC pathogenesis [20], which could be related with our suggestive observations related to the tight junction formation. While other studies, such as Liu et al., were showing its protective effect on UC, since it has significantly attenuated symptoms of DSS-induced colitis in mice [33]. One of the probiotic *Escherichia coli* strains (Nissle 1917) has been shown to be efficient and safe in maintaining remission equivalent to the gold standard mesalazine in patients with ulcerative colitis [34]. However, there are reports, such as Yang et al., showing possible pathological effects of this bacteria in the pathogenesis of UC [6].

Furthermore, our results emphasize the patient-specific nature of the host response to intestinal bacteria, as evidenced by the varied reactions of patients’ colonic epithelial cells to the same bacteria. Therefore, more samples are needed and various stratifications of those to acquire significant and in-depth observations.

## Conclusions

Despite the decreased bacterial diversity and alterations in gut microbiota during UC, a significant portion of these microorganisms are consistently present and remain unchanged throughout the pathogenesis of the disease. Two species - *E. coli* and *P. vulgatus* – belonging to the most stable and unaltered commensal genera of the gut do not cause colonic epithelial stress and are not recognized as pathogens. Nevertheless, both species show a tendency to differentially regulate the tight junction formation in the control- as well as UC patient-derived colonic epithelial cell monolayers.

### Electronic supplementary material

Below is the link to the electronic supplementary material.

Supplementary Material 1

## Data Availability

The 16S rRNA coding gene sequencing data was deposited into OSF database and is available under the accession number: 10.17605/OSF.IO/J3PC6. The qPCR datasets supporting the conclusions of this article are available from the corresponding author upon reasonable request.
