# Supplementary material for: Constituents of stable commensal microbiota imply diverse colonic epithelial cell reactivity in patients with ulcerative colitis
Source: Gut Pathog. 2024 Mar 23;16:16. doi: 10.1186/s13099-024-00612-0 (PMC10960424; doi:10.1186/s13099-024-00612-0)
Supplement: Supplementary file 1 — Supplementary Material 1 [file 13099_2024_612_MOESM1_ESM.pdf]

Supplementary Table 1. Progression of sequencing reads and quality control steps through the DADA2 pipeline.

	Input reads	Filtered reads	Denoised reads	Merged Reads	Non chimeric reads
**Total reads:**	7,430,623	6,696,006	6,489,333	5,717,237	4,824,962
**Per sample**	103,203.097	93,000.08333	90,129.625	79,406.06944	67,013.36111
**Minimum reads**	36,434	32,906	30,722	24,884	22,68
**Maximum reads**	247,909	225,413	223,547	209,297	201,719
**Average reads**	103,203.097	93,000.08333	90,129.625	79,406.06944	67,013.36111

Supplementary Table S2. Differential abundance analysis and relative abundance of genus level comparing control and ulcerative colitis groups. The P values obtained from the Wilcoxon rank-sum test and adjusted using Benjamini-Hochberg (BH) correction to control the false discovery rate. Difference in mean relative abundance were calculated by substracting mean relative abundance of control group from mean relative abundance of UC group.

Genus	statistic	p.value	alternative	p.adjust	mean_abundance_UC	mean_abundance_control	sd_abundance_UC	sd_abundance_control	mean_diff_UC_control
Intestinibacter	613	0,765680255	two.sided	0,875063148	0,022237606	0,008484979	0,090587388	0,008637837	0,013752628
Ligilactobacillus	619,5	0,636994939	two.sided	0,772115078	0,023604952	0,012338923	0,082808148	0,02796755	0,011266028
Faecalibacterium	415	0,041903578	two.sided	0,127486258	0,143654763	0,091973126	0,107639036	0,057358499	0,051681638
Alistipes	848	0,001910204	two.sided	0,019102045	0,011292671	0,041802385	0,018679575	0,086837927	-0,030509714
Phocaeicola	577	0,905819652	two.sided	0,929045797	0,146328678	0,120577352	0,135003828	0,095587877	0,025751326
Escherichia/Shigella	608	0,799152726	two.sided	0,887947473	0,033658088	0,006162883	0,087671059	0,012542362	0,027495205
Prevotella	774	0,023800386	two.sided	0,095201543	0,073085658	0,106632751	0,121152892	0,12037177	-0,033547093
Streptococcus	420	0,04462019	two.sided	0,127486258	0,019641654	0,004911938	0,051298597	0,008447468	0,014729716
Duodenibacillus	510,5	0,234057075	two.sided	0,376554068	0,018133717	0,004058755	0,060403555	0,010522293	0,014074962
Bacteroides	663	0,374703495	two.sided	0,48348838	0,035505637	0,037682229	0,049302715	0,040981994	-0,002176592
Sutterella	509	0,336187153	two.sided	0,448249537	0,039501407	0,02203602	0,059126628	0,035335088	0,017465387
Oscillibacter	765	0,0350811	two.sided	0,116936998	0,016609437	0,017451696	0,039772341	0,021004315	-0,000842259
Collinsella	582	0,952795207	two.sided	0,952795207	0,04927532	0,039194796	0,055507957	0,032634379	0,010080524
Catenibacterium	512	0,28374935	two.sided	0,417827815	0,028819042	0,014871959	0,056982207	0,033319928	0,013947083
Holdemanella	629	0,613191723	two.sided	0,766489653	0,041860455	0,045827583	0,062030328	0,064927249	-0,003967129
Anaerostipes	787	0,018565377	two.sided	0,082512787	0,026794711	0,044526776	0,038088046	0,043505335	-0,017732066
Roseburia	677	0,29247947	two.sided	0,417827815	0,038139761	0,042044186	0,042433335	0,032167931	-0,003904426
Agathobacter	803,5	0,010624622	two.sided	0,059707178	0,030964609	0,051291694	0,041961817	0,044028931	-0,020327086
Faecalibacillus	727	0,092149394	two.sided	0,204776431	0,010773369	0,013167691	0,02535809	0,016478157	-0,002394322
Anaerobutyricum	727	0,100005151	two.sided	0,210537161	0,024501619	0,031326794	0,025134879	0,022041984	-0,006825175
Parabacteroides	714,5	0,133694619	two.sided	0,254656417	0,017648753	0,023662218	0,024550962	0,026794587	-0,006013465
Blautia	680,5	0,2738781	two.sided	0,417827815	0,030983871	0,034214702	0,030036234	0,023817386	-0,003230831
Fusicatenibacter	754	0,049071125	two.sided	0,130856333	0,018930347	0,022793988	0,024968031	0,017656861	-0,003863641
Paraprevotella	880	0,00031784	two.sided	0,004237867	0,006973575	0,013112789	0,018210461	0,011580524	-0,006139214
Mediterraneibacter	933	3,06324E-05	two.sided	0,001225296	0,006621908	0,019071421	0,016047715	0,017360073	-0,012449513
Clostridium_sensu_stricto	667	0,335482411	two.sided	0,448249537	0,006208731	0,008434922	0,011048047	0,015867539	-0,00222619
Romboutsia	618	0,722635276	two.sided	0,850159148	0,014478256	0,017385334	0,013526832	0,017971341	-0,002907078
Clostridium_IV	688	0,119822269	two.sided	0,239644537	0,0025145	0,005575411	0,008402285	0,015590895	-0,003060911
Coprococcus	829	0,004065821	two.sided	0,032526567	0,006925185	0,015025734	0,010851379	0,017038401	-0,008100548
Subdoligranulum	736	0,077675142	two.sided	0,182765039	0,013355039	0,016784602	0,018611162	0,018427682	-0,003429563
Phascolarctobacterium	683	0,186005518	two.sided	0,323487858	0,004672613	0,009423033	0,009146633	0,01645635	-0,00475042
Eggerthella	791	0,011941436	two.sided	0,059707178	0,003874482	0,012300275	0,00695803	0,015043033	-0,008425793
Dorea	770,5	0,03086737	two.sided	0,112244984	0,012346466	0,017819928	0,0084039	0,011509122	-0,005473462
Anaerotignum	602,5	0,841897732	two.sided	0,910159711	0,002115759	0,001786749	0,006163097	0,004232653	0,00032901
Lachnospira	738	0,074427769	two.sided	0,182765039	0,006439452	0,009787383	0,007956116	0,009191687	-0,003347931
Odoribacter	490	0,235346293	two.sided	0,376554068	0,006295407	0,004093278	0,007226709	0,005427589	0,002202129
Ruminococcus2	775	0,010960368	two.sided	0,059707178	0,002056528	0,005746062	0,003951591	0,007505596	-0,003689535
Dysosmobacter	598	0,895787788	two.sided	0,929045797	0,001118504	0,001939318	0,001630924	0,003616566	-0,000820814
Terrisporobacter	688,5	0,176043695	two.sided	0,320079446	0,001659784	0,002730222	0,003406377	0,00371463	-0,001070438
Cuneatibacter	850	0,000220771	two.sided	0,004237867	0,000397688	0,001948113	0,001079893	0,002501489	-0,001550425

Supplementary Table S3. Variance of normalized gene expression values between biological

Condition	Treatment	Gene	Mean	Standard Deviation	Variance
Control	E. coli	HSPA1A	-3,748615742	3,38499027	11,45815913
Control	E. coli	HSPB1	-4,190350533	2,178352381	4,745219094
Control	E. coli	TLR4	-13,03816632	1,626524425	2,645581705
Control	E. coli	ZO1	-11,26916432	2,502080833	6,260408497
Control	P. vulgatus	HSPA1A	-3,945540071	2,0976251	4,400031061
Control	P. vulgatus	HSPB1	-3,993775249	2,409698449	5,806646614
Control	P. vulgatus	TLR4	-11,70871484	3,421294025	11,70525281
Control	P. vulgatus	ZO1	-12,19184371	2,071162055	4,289712257
Control	Untreated	HSPA1A	-4,247148156	1,855312855	3,442185791
Control	Untreated	HSPB1	-4,425023675	1,438194662	2,068403886
Control	Untreated	TLR4	-12,75005233	1,503249895	2,259760247
Control	Untreated	ZO1	-13,08364785	3,00621818	9,037347747
UC	E. coli	HSPA1A	-5,498857922	2,17948001	4,750133114
UC	E. coli	HSPB1	-5,644184536	1,089166983	1,186284716
UC	E. coli	TLR4	-13,81508467	3,912716154	15,3093477
UC	E. coli	ZO1	-12,77051247	4,34003223	18,83587976
UC	P. vulgatus	HSPA1A	-4,738044209	1,627908808	2,650087087
UC	P. vulgatus	HSPB1	-4,826481819	1,03541049	1,072074883
UC	P. vulgatus	TLR4	-13,08008522	1,50476106	2,264305847
UC	P. vulgatus	ZO1	-11,98068545	3,312281908	10,97121144
UC	Untreated	HSPA1A	-5,053045061	0,878625855	0,771983394
UC	Untreated	HSPB1	-5,169733683	0,486062481	0,236256735
UC	Untreated	TLR4	-12,92363972	1,520318062	2,311367011
UC	Untreated	ZO1	-11,51168209	1,987112121	3,948614581
